# Clinical Features and Prognosis of Facial Palsy and Hearing Loss in Patients With Ramsay Hunt Syndrome

**DOI:** 10.7759/cureus.30897

**Published:** 2022-10-31

**Authors:** Ritika Malhotra, Abhay Mudey, Iris Agrawal

**Affiliations:** 1 Medicine, Jawaharlal Nehru Medical College, Datta Meghe Institute of Medical Sciences, Wardha, IND; 2 Community Medicine, Jawaharlal Nehru Medical College, Datta Meghe Institute of Medical Sciences, Wardha, IND

**Keywords:** clinical features, prognosis, hearing loss, facial palsy, ramsay hunt syndrome

## Abstract

Ramsay Hunt syndrome, a late manifestation of varicella-zoster virus infection, occurs when the virus reactivates, infects, and produces an inflammatory reaction in the seventh cranial nerve geniculate ganglion. The detection is made clinically on the basis of the following three features: facial nerve palsy, the presence of characteristic herpetic vesicles around the mouth, and pain in the ear. However, it is often diagnosed quite late and sometimes even missed, increasing the chances of complications that have long-term effects. The two significant complications following the development of Ramsay Hunt syndrome are facial nerve palsy and hearing impairment. Ramsay Hunt syndrome is among the principal causes of facial nerve palsy, implicated in around 2-10% of all cases. While hearing loss though prevalent, is a less common complication than facial palsy.

This review aimed to analyze the clinical presentation and prognostic features of the complications of Ramsay Hunt syndrome mentioned above, that is, hearing loss and facial nerve palsy. It was seen that while the association of Ramsay Hunt syndrome with facial nerve palsy has been studied quite extensively, the literature on hearing loss as a sequela is quite lacking. The course and outcome of facial nerve palsy is determined by the early clinical picture, while the otological symptoms rely on the extent of nerve involvement. Early diagnosis and treatment go a long way in preventing these complications and increasing the chances of complete recovery. Treatment options most commonly studied included antiviral drugs such as acyclovir, steroids, and anti-inflammatory agents.

## Introduction and background

Ramsay Hunt syndrome, also known as herpes zoster otics, occurs when herpes zoster infection affects the facial nerve near one of the ears. Painful rash, facial paralysis, and hearing loss occur in the affected ear. Ramsay Hunt syndrome occurs due to infection by varicella-zoster virus (VZV) [1]. The VZV lives inside the ganglia of spinal and cranial nerves following primary varicella infection. The virus reactivates and then replicates before entering the dermatome connected to the affected ganglion through the sensory nerve fiber. Subsequent viral multiplication in the keratinocytes results in the characteristic clinical signs of herpetiform-distributed vesicles [2]. The vestibulocochlear nerve (CN VIII), which results in hearing impairment, tinnitus, and dizziness, is usually affected in association with the facial nerve (CN VII) [3]. The disease usually presents as herpetic vesicles around the pinna or oral mucosa, otalgia, and facial nerve palsy [4]. The auricle, auditory canal, cheek, or palate can all be affected by erythematous vesicular rash [5]. The prodrome, which frequently lasts for one to three days, includes discomfort, fever, and exhaustion, and is preceded by the occurrence of facial paralysis [6]. The appearance of a painful red rash with fluid-filled blisters might occur before or after the palsy [7]. Ramsay Hunt syndrome has a wide range of side effects on patients, with the acute stage being characterized by pain, paralysis, cochleovestibular symptoms, and mental health issues [8]; idiopathic non-traumatic facial paralysis is most frequently seen in people who have Ramsay Hunt syndrome. Taking into account features such as facial paralysis, otalgia, and herpetic lesions with a cranial dermatome, 12% (185) of the patients in a longitudinal study conducted on 1,507 individuals suffering from facial palsy on one side had Ramsay Hunt syndrome [9]. Around 2-10% of all occurrences of facial paralysis are brought on by Ramsay Hunt syndrome. Paresis or total paralysis may occur in patients, with the latter group having the worst prognosis for recovery. Few people with this syndrome continue to have complete facial paralysis, but around half of them still have some facial motor impairment [9-11]. Even in one-third of cases of Ramsay Hunt syndrome, blisters appear preceded by facial paralysis, making it challenging to diagnose Ramsay Hunt syndrome at the time of onset of facial paralysis [3]. Bilateral signs, many inclusions of other cranial nerves, delayed progression, insufficient recovery within three months of initiation, or systemic symptoms raise the possibility of a nonviral etiology [12].

The following review describes the clinical picture and the predictive features of facial palsy and hearing impairment as sequelae of Ramsay Hunt syndrome.

## Review

Prevalence

The annual frequency of Ramsay Hunt syndrome is grossly 5 per 100,000 persons and can affect equally both immunocompetent and immunocompromised patients [13]. Around 7% of total acute cases of facial paralysis result as a consequence of Ramsay Hunt syndrome, while in 10% of those, zoster sine herpete is implicated [14].

Risk factors

Immunocompromised patients are more susceptible to developing complications and have a more severe clinical picture. Anyone can get Ramsay Hunt syndrome, although people in their seventh and eighth decades of life are most vulnerable [7]. Risk factors for Ramsay Hunt syndrome reflect those that increase the chances of contracting a VZV infection, such as immune deficiency, chemotherapy, stress, and nutritional deficiencies, among others [8].

Facial nerve paralysis

Bell's palsy is a condition that causes a temporary weakness of the facial muscles on the affected side resulting from a dysfunction of the facial nerve. Out of the five extratemporal branches of the facial nerve, the zygomatic branch, which is responsible for supplying the zygomaticus major muscle, crucial for smiling, is particularly important to people who intend to use it for facial paralysis. Facial paralysis primarily results from Bell’s palsy and Ramsay Hunt syndrome. Recent findings show that an infection caused by a virus leads to Bell's palsy [15-17]. The VZV infection in Bell's palsy patients should be confirmed because Bell’s palsy, which results in peripheral facial paralysis, is believed to be a consequence of herpes simplex virus (HSV) activation. The other virus, most frequently linked to Bell's palsy, is VZV [18]. The seventh nerve mostly innervates the muscles that move the face, with some sensory nerve fibers carrying taste sensations from the taste receptors on the anterior two-thirds of the tongue. Facial weakness on the affected side in those suffering from Ramsay Hunt syndrome is evident as inability to wrinkle the forehead, close the eyes, and grin without symmetry. When instructed to grin or expose their teeth, the affected side cannot do so, and the upper axis shifts to the unaffected side. Additionally, decreased muscular facial muscular control causes saliva and tears to flow less freely on the side that is afflicted. Lack of ability to close the eye causes excessive eye dryness, which can cause cataract ulcerations [2,19].

There are various ways to assess the injury in facial paralysis. Historically, the site of the lesion was diagnosed using topodiagnostic tests that made use of facial nerve and its branches. These tests may be done but they are not currently in use and are of historical value. The tests are the stapedial reflex test, lacrimation test, and electrogustometry test [20]. Nerve excitability tests can also be performed for facial nerve paralysis. These mainly determine the changes in the excitation threshold [21]. Ramsay Hunt syndrome makes VZV's involvement in facial paralysis evident, but there are still unanswered questions about HSV type 1 (HSV-1)'s involvement in Bell's palsy, such as do the two viruses insidiously coexist within the geniculate ganglion at the prevalence outlined by Furuta et al. [22].

In Ramsay Hunt syndrome, zostiform vesicles on pinna that contain contagious VZV particles and serological confirmation of persistent VZV infection accompany facial paralysis. The disorder resembles a peripheral sensory ganglia-like reactivation of the virus in terms of classic VZV, which is corroborated by elevated anti-VZV IgM antibody titer in these people [23]. Bell's palsy cannot be accurately diagnosed by serum tests for growing herpes virus antibody titers. For HSV-1 or herpes zoster, salivary polymerase chain reaction (PCR) is being used for the detection of the virus during its multiplying phase; however, these assays continue as a research tool [24]. Additionally, VZV is expected to be the cause of Bell's palsy in a subset of patients who show serological data of asymptomatic VZV reactivation but no cutaneous deformities [25,26]. Mumps, Epstein-Barr virus (EBV), cytomegalovirus, rubella, and, in recent years, human immunodeficiency virus have also emerged as potential agents, which can be occasionally linked to Bell's palsy [27-31]. Different studies found different rates of full recovery rates depending on the kind of steroid linked to acyclovir [32,33].

It makes sense that antiviral medications could benefit those who have Ramsay Hunt syndrome, which causes facial paralysis. Other viral infections within the body are frequently treated with these medications. Trials that might solve this issue are very limited in number; therefore, their value is somewhat questionable. When deciding whether to use these medications for Ramsay Hunt syndrome, the risks of doing so must be weighed against the uncertain possibility of benefit. This is because patients who take these medications may encounter side effects [34]. Antiviral medications are typically used as the initial course of treatment for herpes zoster infections at different sites on the body and are believed to decrease or prevent nerve damage, improving results. These medications may increase the likelihood that facial weakness in Ramsay Hunt syndrome patients may get better or go away entirely [34]. Administration of steroids may help in decreasing facial nerve inflammation, and surgery might alleviate facial nerve compression, while acyclovir has been found to considerably treat the putative inciting infection [35].

Prednisone and acyclovir, if administered timely, appear to enhance the prognosis, according to evidence collected from case reports and retrospective studies, in spite of the shortage of randomized controlled trials for Ramsay Hunt syndrome [36]. Clarifying the etiology of Bell's palsy may need looking at how anti-HSV-1 medication affects this syndrome. Given that steroids have a somewhat positive effect on Bell's palsy [37], acyclovir was given with prednisone for the treatment of this ailment in order to answer this question [38]. A retrospective study on the treatment of the Ramsay Hunt syndrome [13] revealed a statistically remarkable recovery in patients who got acyclovir and prednisone within three days of the start of the disease. Treatment likely works better within the first 72 hours but less effectively after seven days [24].

We come to the conclusion that corticosteroids combined with antiviral medicine form a significant contributing role in enhancing facial nerve activity recovery in cases of Ramsay Hunt syndrome [39]. Decompression surgery, which involves removing the bone which is constricting the facial nerve, is occasionally carried out for patients who may not fare well, as determined by a range of clinical tests, such as low House-Brackmann scores and more than 90% facial nerve deterioration, as shown by electroneurography (ENoG). House-Brackmann is the grading system for paralysis and ENoG as the test, likely used for prognostication. This surgery needs to be done in less than 14 days from the onset of total paralysis [40]. However, additional surgery is the last measure in patients with a good outlook since it is an invasive procedure and because it has been linked to problems such as ipsilateral deafness, CSF leakage, and convulsions. Since only those with poor outcomes and a definite need for surgery should undergo a decompression procedure, early but accurate prognosis prediction is crucial [35,24]. Symptomatic care is essential, especially for the painful elements of Ramsay Hunt syndrome. With zoster, analgesia is frequently required; long-acting opioids, acetaminophen, or ibuprofen can all be administered [8]. Although the shingles vaccine is not completely effective, it is likely to prevent Ramsay Hunt syndrome [41].

**Figure 1:**
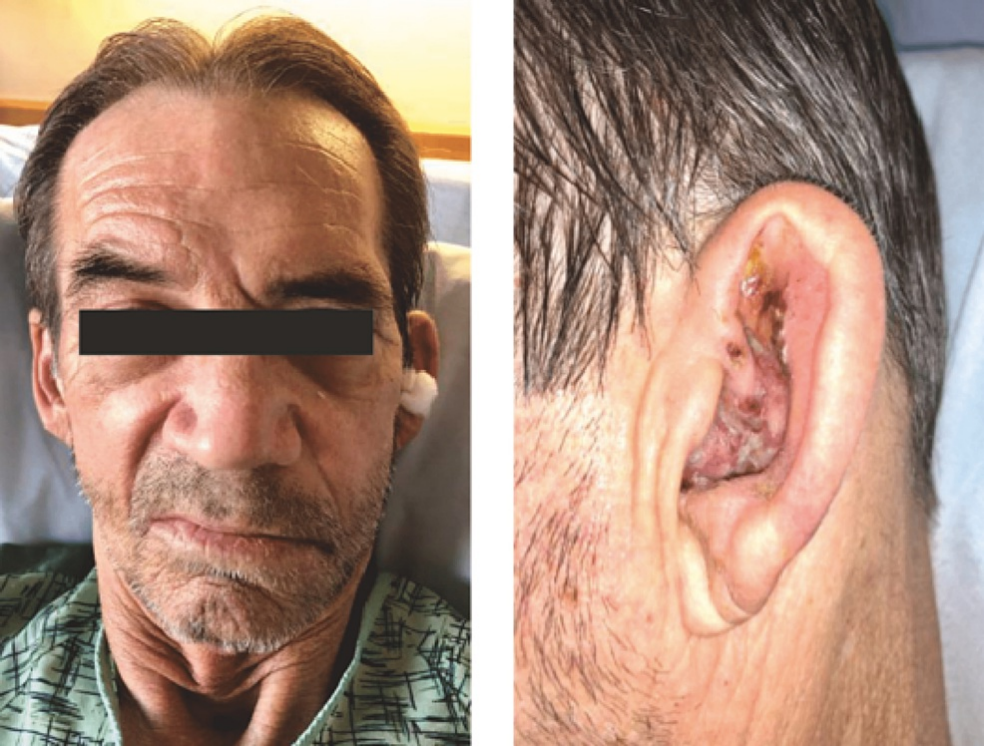
Left: facial droop. Right: vesicles on pinna. Open access journal under a CC-BY license contributed by Letchuman V, Donohoe CD. Brainstem and cerebellar involvement in Ramsay Hunt syndrome. Case Rep Otolaryngol. 2019;2019:7605056. doi: 10.1155/2019/7605056

The majority of patients, almost 85%, recover partially within a time span of three to four weeks and fully in six months. Patients who initially suffer partial paresis have a 93-98% likelihood of fully recovering. Complete paralysis, patients aged 60 years and above, minimal improvement after three weeks, pregnancy, nerve atrophy with electrophysiological assessment, and underlying diseases such as diabetes mellitus all point to a poor prognosis [42]. The patient's social life is significantly impacted by permanent facial disfigurement and other potential Ramsay Hunt syndrome complications. To prevent these consequences, it is crucial to diagnose and treat this at an early stage [43]. The above findings can be seen in Figure 1.

Hearing loss

Hearing loss can manifest as a consequence of various pathologies associated with Ramsay hunt syndrome. Despite the fact that the manifestations of the Ramsay Hunt syndrome in patients have been well studied, a thorough evaluation of the audiological data and its correlation to clinical manifestations and the outcome for hearing impairment are not thoroughly investigated [44]. When reported for geniculate neuralgia, sensorineural hearing loss (SNHL) has a documented incidence of 22% after microvascular decompression (MVD) [45]. Drill-induced noise or a brief loss of CSF fluid during MVD for the geniculate ganglion may be the causes of high-frequency hearing loss that affect the ipsilateral side the most [45]. Following middle ear surgery, delayed facial palsy (DFP), a rare complication that rarely lasts longer than 72 hours, can develop. The risk of VZV reactivation should be raised if acute otalgia occurs before DFP [46]. After the progression of VZV, cochlear neuritis might result in persistent hearing loss in Ramsay Hunt syndrome patients [47]. Ramsay Hunt syndrome patients experience vestibulocochlear symptoms as the vestibulocochlear, CN VIII, is close to the geniculate ganglion and is irritated by transneuronal reinfection from VZV [47]. Hearing loss and vertigo can all be brought on by the vestibulocochlear nerve's close closeness to the facial nerve. In Coulson's series, patients with tinnitus (20%), imbalance or vertigo (51%), and SNHL (43%) were also present [8].

In a retrospective study conducted on the patients in the ENT division of Rabta University Hospital Centre in Tunisia, 15 of the 30 Ramsay Hunt syndrome patients treated over a seven-year period (2003-2009), with an average age of 46 years and consultations made on average eight days after symptom start, were included in retrospective research. During the days before their symptoms began, four patients were exposed to cold. In each instance, facial asymmetry together with a conchal vesicular rash was the primary presenting symptom. There was concomitant otalgia in 13 individuals. Nine had some hearing loss, and four had rotational vertigo and unsteady walking. With an average threshold of 55 dB, systematic audiography demonstrated unilateral perceptual hearing loss in 11 cases (range: 30-90 dB). In each case, the stapedial reflex was lost on the same side of the facial palsy [48]. Others have noted the occurrence of cochlear and retrocochlear types of hearing loss. In a study conducted by Herbert and Young, a patient presenting with impaired speech discrimination, no recruitment by short increment sensitivity index, and a positive tone decay showed no signs of recovery [49]. Autoimmune illnesses can present as bilateral sudden SNHL and recurrent facial nerve palsy, pursuant to the case study conducted by Psillas et al. A syndrome similar to Ramsay Hunt syndrome resulting from an HSV infection may be the initial cause [50]. VZV may have entered the labyrinth by an open canal of facial nerve, consequently crossing the oval and circular windows in Ramsay Hunt syndrome patients' middle ear mucosa [51]. Direct neuronal transmission is another possibility, although a PCR study on the spread of HSV-1 could not find any evidence to suggest emigration of HSV-1 along the vestibulofacial anastomosis [52]. However, the same researchers were able to show that a VZV genome was present in geniculate ganglion and in addition to this also seen in the spiral and vestibular ganglions, by employing PCR [53]. A frequent cause of unilateral partial vestibular paralysis that typically retains posterior semicircular canal function is vestibular neuritis. HSV-1, a latent form of the double-stranded DNA, HSV, is thought to have been reactivated by a virus in the vestibular ganglia [54]. Herpetic facial paralysis has long been known to be accompanied by auditory abnormalities. The herpes zoster virus should be investigated in patients who have SNHL along with idiopathic facial paralysis, even with deficiency of vesicles.

Age 64 or younger is one factor that favors the recovery of hearing functions. Restoration of auditory function does not occur, but high-tone SNHL may last, unless the patient is extremely young [11].In cases of acute hearing loss and facial paralysis, vertigo has been linked to a bad prognosis. Vertigo appears to serve as a helpful prognostic factor when it is absent [11]. A case report concluded that chronic otalgia might be a kind of post herpetic neuralgia after Ramsay Hunt syndrome, with complications involving the cervical nerve while there are no skin lesions in the cervical region. Ramsay Hunt syndrome-related refractory otalgia may be treated with pulsated radiofrequency to the great auricular nerve [54].

## Conclusions

Ramsay Hunt syndrome, also commonly referred to as herpes zoster oticus or geniculate ganglion herpes zoster, is a consequence of reactivation of VZV infection. The most common clinical presentation includes the triad of facial paralysis, ear pain, and zosteriform vesicles around the ear, generally on the auditory canal. Facial palsy and hearing loss are the disease's most severe and frequent manifestations, interfering with the patient's daily activities and decreasing the quality of life. The patient may end up in a debilitating condition.

Bell's palsy and Ramsay Hunt syndrome are the two significant diseases leading to facial nerve paralysis around the world. Although Bell's palsy is the leading cause, it's prognosis in case of facial nerve paralysis is slightly better than Ramsay Hunt syndrome; thus, it is imperative to differentiate between the two in a patient presenting with facial nerve paralysis. The symptoms were identified to be decreased or absence of wrinkling of the forehead, weakness of the afflicted side of the face, sagging of mouth on the ipsilateral side, loss of ability to close the eye, and muscular weakness leading to dryness in the eyes and mouth. Facial nerve paralysis is the most critical indicator for the prognosis of Ramsay Hunt syndrome. Hearing loss may result as a form of cochlear neuritis developing in Ramsay Hunt syndrome. Timely diagnosis and prompt treatment with antivirals and steroids such as acyclovir and prednisolone have provided a better prognosis.
